# Microplastic Index—How to Predict Microplastics Formation?

**DOI:** 10.3390/polym15092185

**Published:** 2023-05-04

**Authors:** Arjen Boersma, Kalouda Grigoriadi, Merel G. A. Nooijens, Sieger Henke, Ingeborg M. Kooter, Luke A. Parker, Ardi Dortmans, Jan Harm Urbanus

**Affiliations:** 1 TNO, HTC 25, 5656 AE Eindhoven, The Netherlands; 2 TNO, Princetonlaan 6-8, 3584 CB Utrecht, The Netherlands

**Keywords:** microplastics, polymer properties, impact, wear, MPI

## Abstract

The presence of microplastics in environmental compartments is generally recognized as a (potential) health risk. Many papers have been published on the abundance of microplastics at various locations around the globe, but only limited knowledge is available on possible mitigation routes. One of the mitigation routes is based on the choice of plastic materials used for products that may unintentionally end up in the environment. As a first approach, this paper presents a method to calculate the tendency of polymers to form microplastics, based on their mechanical and physical properties. A MicroPlastic Index (MPI) that correlates the microplastic formation to polymer properties is defined for both impact and wear of polymers via a theoretical particle size and the energy required to form these particles. A first comparison between calculated and experimental particle size is included. The MPI for impact and wear follow the same trend. Finally, these MPIs are correlated to the respective abundance of the microplastics in the environment, corrected for global production of the corresponding polymers: the higher the MPI, the more microplastics are found in the environment. Thus, the MPI can be used as a basis for choice or redesign of polymers to reduce microplastic formation.

## 1. Introduction

Microplastics are a growing concern for the implementation of polymer materials in a circular economy. According to the European Chemical Agency (ECHA), microplastics are defined as a material composed of solid polymeric-containing particles, to which additives or other substances may be added, having sizes below 5 mm [1]. Environmental and health effects of microplastics have been published in many papers [2,3,4,5,6] and the presence of microplastics has been shown at many (unexpected) locations [7,8,9,10,11]. All types of polymers have been found in the environment, in a large range of particle sizes. For the mitigation of microplastics, several solutions have been proposed, ranging from banning intentionally added microplastics from cosmetics [12], to the removal of plastics waste materials from rivers and oceans as proposed and demonstrated by The Ocean Cleanup [13], and other governance mitigation measures [14].

A few researchers have correlated polymer properties with degradation and microplastic formation. Min et al. [15] used hydrophobicity, crystallinity and glass transition temperature to predict the plastic degradation into microplastics. Yuan et al. [16] ranked the potential hazards of microplastics in a marine environment, based on global production, potential degradation and experimental particle size. However, to our knowledge, there is no study that correlates the formation of microplastics directly to the basic properties of the polymers. The same applies for microplastic formation from mechanically or chemically recycled plastics, whose properties may deviate from the virgin properties and thus have a different tendency to form microplastics. An interesting approach towards the mitigation of microplastics formation is the assessment of individual polymers for their tendency to form microplastics based on polymer properties, that may differ between the various polymer types and grades, and will change during ageing and degradation in the environment. It is important to establish this correlation between polymer properties and microplastic formation for two reasons: (1) the toxicology of the microplastics may depend on the type of plastics, due to both physical and chemical interactions, and thus identification of the polymers with the highest contribution to microplastic formation will help the toxicological understanding of the problem; (2) to reduce the release of microplastics into the environment, it is essential to understand which polymer has the highest contribution, and how we can mitigate this. By knowing the correlation between polymer properties and microplastic formation, polymer producers and users may adapt the plastic behavior and as such help to reduce the problem. In addition, the future removal of microplastics from the environment may be improved by a better understanding of the types of plastics emitted to the environment. A better insight in the relation between polymer type and microplastic formation may assist in this mitigation approach.

It has to be emphasized that most of the microplastic issues presently found in the environment pollution and health concerns are caused by petrol-based non-biodegradable polymers. Microplastics formed from biodegradable polymers will be relatively quickly converted into CO_2_ and water by the microorganisms in the soil and water, and will therefore pose a much lower environmental and health risk.

In this paper we present a microplastic formation model, based on several mechanical and physical polymer properties of which impact strength (determining crack growth) and wear resistance (relevant to abrasion) are among the most important. These two parameters are expected to be the most relevant in polymer recycling processes and wear and tear of polymers in the environment. By using these parameters, critical dimensions in breaking polymers can be predicted that are an indication for the size of the microplastics that are formed by means of impact, friction etc., when external stressors are applied to the material, by means of wind, water or soil abrasion. This approach generates a Microplastic Index (MPI), being an indication for the tendency of polymers to form microparticles when exposed to mechanical energy. A high MPI indicates a high tendency towards the formation of microplastics. The MPI is constructed from the expected particle size that is generated from the plastic products and the energy that is needed to create these particles. This MPI can be used to compare (a) polymer types and (b) polymer grades, and as such leads to decisions on mitigation measures. 

Since polymer properties are often affected by ageing and degradation, the MPI is affected as well. As such, ageing and degradation generally lead to a higher MPI, indicating higher amounts of microplastics formed. To demonstrate the approach and underlying physical assumptions, the MPI calculations are made for 14 virgin polymers for which the mechanical properties can be found in databases such as www.matweb.com. In addition, some properties cannot be recovered from these databases, and need to be obtained from experimental correlations or from additional experiments.

The starting point of this study is that when plastics form microplastics, fracture mechanics modelling can predict a typical size of particles formed. We implement established fracture mechanics relations to derive the correlation between particle formation and polymer properties. This has already been demonstrated for, e.g., glass and ceramics [17], and we convert this approach here to polymers. The MPI is calculated for two cases: impact fracture and wear. The fracture mechanics for these cases is different and are treated separately. A first validation of the calculated particle sizes was experimentally done using three different polymers (HDPE, PP, PET, PS).

The development of the MPI is demonstrated for bulk polymer products in which the material properties are isotropic, and the fracture behavior is equal in all direction. In a forthcoming paper, we will extend the MPI model to anisotropic products, such as textiles and fibers, that may have a different fracture mechanism. The use and washing of textile are generally seen as a major source of microplastics in the environment, and is therefore also an important contribution to the microplastic problem.

The layout of the manuscripts is as follows: First, some experiments are presented to demonstrate the difference in microplastic formation behavior of four types of polymers (HDPE, PP, PET and PS) when these are exposed to the two fractionation steps, grinding and sanding, and to show the necessity for the development of a theoretical base of understanding this behavior (Section 2). Then, a comprehensive summary of the impact (Section 3) and wear (Section 4) mechanisms is given resulting in the derivations for the new MPI models for both cases, which enables the prediction of the microplastic formation behavior. This is followed by the assessment of the MPI models for 14 virgin polymers, by using average literature data (Section 5 and Section 6). Finally, the impact and applicability of the MPI is demonstrated by comparing the MPI and the relative abundance of microplastics found in the environment for each polymer (Section 7). Thus, showing how the MPI can aid microplastics understanding on the global scale.

## 2. Experimental Evidence of the Difference in Microplastic Formation Depending on Type of Polymer

**Figure 1:**
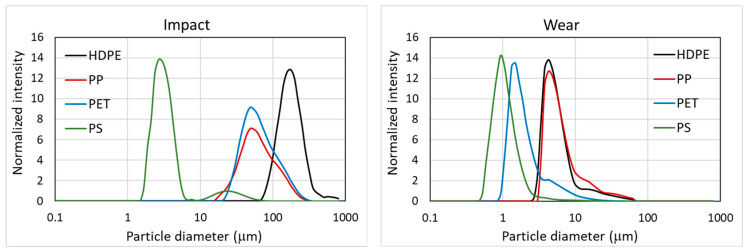
Experimental number particles size distributions of the four polymers considered in the validation (HDPE, PP, PET, PS).

An experimental microplastic formation study is presented here for four polymers: high density polyethylene (HDPE, blow molding grade, LyondellBasell, Ferrara, Italy), polypropylene (PP, homopolymer, LyondellBasell), poly-ethylene terephthalate (PET, bottle grade, Dufor, Zevenaar, The Netherlands) and polystyrene (PS, Nr 182427, MerckSigmaAldrich, Amsterdam, The Netherlands). The polymers are reduced in size using two techniques, (1) milling in a Retsch ZM100 centrifugal mill to simulate impact fracture, and (2) sanding on a Struers (Cleveland, OH, USA) Knuth-Rotor 2 polishing machine with SiC sanding paper to simulate abrasion and wear. The centrifugal mill is equipped with different mesh filters (80–500 μm). It is expected that the smallest particles are generated using the smallest mesh. HDPE, PP and PET were separated using a 250 μm mesh whilst for PS a 120 μm mesh was used. Smaller meshes induced melting and agglomeration of the starting material, preventing the formation of relevant amounts of material to be tested. The sanding machine can be operated with different grit sizes (ISO P500-P2000). The experiments were performed with a grit size of P1000 as larger grits did not yield the smaller particles and smaller grits resulted in melting of the polymers. After milling and sanding, the particles are collected and characterized for their particle size distribution using a Shimadzu (Kyoto, Japan) SALD7500 nano Static Laser Scattering analyzer. The particles are dispersed in 1-propanol, to ensure good wettability of the liquid and dispersion of the particles. For each of the experiments, a distribution of particle sizes is generated. The average particle size decreases when smaller mesh filters or finer grits are used. However, it was found that the smallest particles in each experiment have a more or less constant size, independent of the mesh or grid size, and determined by the material properties. For the comparison of the polymers, the smallest particle sizes that are produced in relevant quantities during the processes of milling and sanding are compared. We do not want to include the very low amounts of small particles that may accidentally be formed by variations in the processes or defects in the materials. Therefore, the characteristic particles sizes are derived from the number average particle size (=D [1, 0]) as calculated from the particle size distribution given by the Static Laser Scattering experiments [18]. The number particle distributions are shown in Figure 1.

The number average particle sizes are calculated from the size distributions of Figure 1. These experimental values are listed in Table 1, The measured values include standard deviations that were calculated from the measured particle size distribution.

The experimental results presented in Table 1 indicate that both the type of polymer and the type of mechanical stress applied to the polymer have a significant influence on the size of the resulting microplastics. More ductile polymers form larger particles; more brittle polymers smaller particles. For the prediction of this behavior, it is therefore important to derive models that incorporate these differences. In the following sections, we will present the microplastic formation models for both impact and wear.

## 3. Derivation of MPI for Impact

Plastic materials that are processed in automated packaging machine, used in everyday life or shredded during recycling, may suffer from impact damage leading to the formation of microplastics. This section describes the formation of microplastics from impact stresses.

### 3.1. Dugdale Model for Size of the Plastic Zone during Impact Fracture

Microplastics are formed when a plastic product is exposed to external stresses, such as wear and friction, or impact, tensile and bending stresses. Additionally, internal stresses in a product can cause the formation of microparticles. These can occur during swelling or shrinkage of (part of) the product. The size that these plastics particles can attain is determined by the properties of the material. In the case of polymers, failure almost always happens via a brittle-ductile failure mechanism. Tough polymers fail under stress via a ductile failure process, in which a large plastic deformation occurs. Brittle materials fail because the applied stress introduces crazes and cracks that is followed by rapid crack growth and failure. Most polymers show a combination of the two failure modes: first the formation of a plastic zone from which a brittle crack can grow.

**Figure 2:**
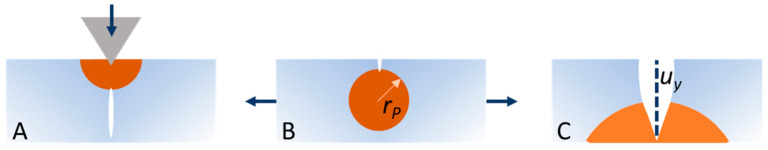
Cracking under indentation load (**A**); cracking under tensile load (**B**); Crack tip opening displacement (**C**). Orange is the plastic zone, r_p_ is the radius of this zone, and u_y_ the crack tip opening displacement.

In a plastic sample that is loaded in impact or indentation (Figure 2A) or tensile (Figure 2B) at a stress that causes failure, a plastic zone is created in which part of the energy is dissipated. For brittle polymers this plastic zone is very small, and the polymer fails below the yield stress. For ductile polymers, this plastic zone is much larger and may even penetrate throughout the thickness of the sample, thus causing full ductile failure. The theoretical particle size that can be formed during failure of a plastic is derived from this principle. The theory of Linear Elastic Fracture Mechanics has been proposed by several authors, each having a slightly different starting point or set of assumptions or boundary conditions. Dugdale [19] and Irwin [20] were among the first to describe the fracture of ductile polymers. They differentiated the plastic zone diameter in front of a crack (r_P_) and the critical crack tip opening displacement (CTOD) (δ_C_), being the size of a crack under stress before further failure. Other authors expanded the theory, or derived similar equations, e.g., Barenblatt [21,22], Bilby-Cottrell-Swinden [23], Huang-Guo [24]. Many handbooks on fracture mechanics summarize the theoretical description of the formation and crack growth (e.g., Antolovich [25] or De With [26]). A clear summary was given by Xu [27]. In this paper, we only propose the Dugdale-Barenblatt and Irwin models to show the similarity of these equations. The radius of the plastic zone as proposed by Dugdale and Irwin is quite similar (Moore [28]):

(1) $$r_{\mathrm{DB}}≅\frac{{\pi K}_{\mathrm{IC}}^{2}}{16\sigma_{Y}^{2}}\;\;\;\;\;\mathrm{Dugdale-Barenblatt}$$

(2) $$r_{I\sigma }≅\frac{K_{\mathrm{IC}}^{2}}{2{\pi \sigma }_{Y}^{2}}\;\;\;\;\;\mathrm{Irwin for plane stress}$$

(3) $$r_{I\varepsilon }≅\frac{K_{\mathrm{IC}}^{2}}{6{\pi \sigma }_{Y}^{2}}\;\;\;\;\;\mathrm{Irwin for plane strain}$$

K_IC_ is the critical stress intensity factor, and σ_Y_ the yield strength. During cracking, the plastic zone is deformed at a critical CTOD at which the crack starts to grow. The CTOD can be calculated from the displacement behind the effective crack tip, according to (Kerkhof [29], De With [26], Xu [27]):

(4) $$u_{y}=\frac{\kappa +1}{2G}K_{\mathrm{IC}}\sqrt{\frac{r_{P}}{2\pi }}$$

where, G is the shear modulus (G = E/2(1 + ν)), E is the Young’s modulus, ν is the Poisson’s ratio and is correlated to κ as:

(5) κ=(3−ν)/(1+ν)     Plane stress

(6) κ=3−4ν     Plane strain

The displacement zones can now be calculated from the crack tip opening (δ_C_ = 2 u_y_) and the size of the plastic zones for the three cases:

(7) $$\delta_{\mathrm{DB}}≅\frac{\sigma^{2}\pi a}{\sigma_{Y}E}=\frac{K_{\mathrm{IC}}^{2}}{\sigma_{Y}E}\;\;\;\;\;\mathrm{Dugdale-Barenblatt}$$

(8) $$\delta_{I\sigma }≅\frac{4\sigma^{2}\pi a}{{\pi \sigma }_{Y}E}=\frac{4K_{\mathrm{IC}}^{2}}{{\pi \sigma }_{Y}E}\;\;\;\;\;\mathrm{Irwin for plane stress}$$

(9) $$\delta_{I\varepsilon }≅\frac{4\left(1-\nu^{2}\right)\sigma^{2}\pi a}{\sqrt{3}{\pi \sigma }_{Y}E}=\frac{4\left(1-\nu^{2}\right)K_{\mathrm{IC}}^{2}}{\sqrt{3}{\pi \sigma }_{Y}E}\;\;\;\;\;\mathrm{Irwin for plane strain}$$

The Dugdale-Barenblatt values are similar to the plane stress and plane strain values of the Irwin model. When the samples are very thin, such as packaging films, the situation of plane stress occurs. For very thick samples, the formulas of plane strain apply. The Dugdale equations resemble the plane stress conditions and are valid for thin samples. The assumption for the minimal microplastics size, is that the particle that will break from a plastic fragment cannot be smaller than the CTOD (=u_y_) [29,30,31]. The plastic zone first deforms to a displacement of δ. If this zone is smaller than u_y_ (or δ_DB_/2), then all energy is converted into plastic deformation and no additional crack surface is formed, that causes the material to fragment further. This means that the critical particle size of polymer particles that can be generated by impact fraction is related to the Dugdale-Barenblatt equation according to:

(10) $$\delta_{I}=\frac{\delta_{\mathrm{DB}}}{2}≅\frac{K_{\mathrm{IC}}^{2}}{2\sigma_{Y}E}$$

Only a few authors have used this approach for the assessment and prediction of the size of (plastic) particles. Schmidt [30,31] compared the Dugdale-Barenblatt equation with experimental results for PS and PEEK during wet grinding. Wolff [32] ground poly(amide imide) and also found comparable particle sizes as predicted by Equation (10). An interesting review paper (Xu [27]) uses the Dugdale theory to describe the fracturing of clayey soils. To our knowledge, no study has been published regarding the fracture mechanics theories in the assessment of microplastics in the environment. Thus, we present a new approach to predict the particle size that can be expected, when polymer products start to wear and tear in the environment.

### 3.2. Energy Dissipation

The energy required for the fracture of polymer materials is often described by the J-integral. De With [26] and Xu [27] summarized this approach. The J-integral describes the change in potential energy due to crack propagation. It avoids the direct calculation of elastic-plastic deformation and stress near the crack tip, but follows a far field contour around the crack. The J-integral has a physical meaning as it is equivalent to the energy release rate (Gr) of the crack when it grows in the x-direction per unit length, as was derived by Griffith [33] and can be calculated from the stress intensity factor and the Young’s modulus:

(11) $$J=\mathrm{Gr}=\frac{K_{\mathrm{IC}}^{2}}{E}=\sigma_{Y}\delta_{I}$$

This is the energy that is required to create crack surface in J/m^2^. δ_I_ is the particle size as calculated in the previous section according to the DB model for the impact case. However, not all energy applied to a polymer is converted into cracking; a significant part is lost as elastic energy and subsequent heating. An estimation of the lost energy can be derived from an impact test, such as a Charpy notched (C_N_) test that is generally used for the assessment of impact strength [34]. The result of a Charpy test is given as energy per area of fractured material (J/cm^2^). This includes the energy required to create and propagate the crack, (i.e., energy release rate) but also the energy for elastic bending etc, that is converted to heat. The ratio of energy release rate (Gr) and Charpy impact energy (C_N_) is an estimate of the fraction of the energy(ξ) that leads to fracture:

(12) $$\xi =\frac{\mathrm{Gr}}{C_{N}}=0.1\frac{K_{\mathrm{IC}}^{2}}{{\mathrm{EC}}_{N}}$$

In which K_IC_ is in MPa√m, E in GPa and C_N_ in J/cm^2^.

### 3.3. Microplastic Formation Index from Impact

Assuming that the fraction ξ of the energy applied to a polymer sample is leading to cracks, and the generated particles are spherical, it is possible to calculate the volume and number of particles that are generated when a stress is applied. The volume and number of particles per surface area of a particle with diameter δ_I_ are respectively:

(13) $$\frac{V_{\delta }}{A_{\delta }}=\frac{\frac{4}{3}\pi {\left(\delta_{I}/2\right)}^{3}}{4\pi {\left(\delta_{I}/2\right)}^{2}}=\frac{\delta_{I}}{6}$$

and

(14) $$\frac{N_{\delta }}{A_{\delta }}=\frac{1}{4\pi {\left(\delta_{I}/2\right)}^{2}}=\frac{1}{{\pi \delta }_{I}^{2}}$$

The volume and number of particles with diameter δ_I_ per J introduced energy then becomes:

(15) $$V_{\delta }=\xi \frac{1}{\sigma_{Y}\delta_{I}}\frac{\delta_{I}}{6}=\frac{\xi }{6\sigma_{Y}}$$

and

(16) $$N_{\delta }=\xi \frac{1}{\sigma_{Y}\delta_{I}}\frac{1}{4\pi {\left(\delta_{I}/2\right)}^{2}}=\frac{\xi }{{\pi \sigma }_{Y}\delta_{I}^{3}}\;\;(\#/J)$$

The total volume of plastic particles that is generated does not seem to depend on the particle size of the generated particles, but only on the yield strength and energy ratio ξ. Thus, the lowest volume of fractured material will be generated from plastics with a high yield strength. The number of particles that are produced does depend heavily on the size. Plastics with poor impact properties may produce high numbers of microplastic particles in the micron range. This value is an indication of the tendency of plastics to form microplastics. If the plastics form small particles, then this number will be larger, whereas when it only forms larger fragments, this number will be smaller. We will use this number in the assessment of plastics and will introduce the MicroPlastic Index (MPI) as the logarithm of the number of particles N_δ_, normalized for a standard number of particles of N_REF_ = 1/J:

(17) $${\mathrm{MPI}}_{I}=\log\left(\frac{N_{\delta }}{N_{\mathrm{REF}}}\right)=\log\left(\frac{\xi }{{\pi \sigma }_{Y}\delta_{I}^{3}}\right)$$

## 4. Derivation of MPI for Wear

Plastic products littered in the environment or moved around in use may show wear damage, because of rubbing against other materials. Also, grinding or sanding of polymers by a grinding wheel or sanding paper induces wear, which results in a different deformation and fracture mechanism in the processed materials than impact fracture.

### 4.1. Critical Depth for Fracture

Similar to impact failure, wear damage can also occur via ductile or brittle abrasion mechanisms. The MPI for wear is determined by a critical depth of penetration of abrasion that determines the final particle size. This is not necessarily the ductile-brittle transition depth, as we will show in this section. Several papers discuss the transition between a ductile and brittle grinding regime, even for brittle materials, based on grinding load and depth of penetration of the abrasive particles (Bifano [35], Lawn [36], Brinksmeier [37], Wu [38]). If the shear stress exceeds the yield strength of the polymers, the mechanism of deformation will move from reversible energy storage, via elastic/plastic elongation to irreversible energy dissipation. The latter can occur via a ductile or a brittle failure regime. Bifano [35], Marshall [39] and Brinksmeier [37] presented a model to calculate the transition depth between ductile and brittle grinding. They state that when the penetration depth of the abrasive media is smaller than this critical depth, the deformation mechanism is ductile. If the penetration depth is larger, then brittle failure occurs. The Bifano-Marshall-Brinksmeier model is based on the critical depth for fracture during indentation of hard materials, combined with the Griffith energy for crack propagation. Using a set of brittle ceramics with various hardnesses (H), the relation between critical depth and material properties was established:

(18) $$d_{C}=0.15\left(\frac{E}{H}\right){\left(\frac{K_{\mathrm{IC}}}{H}\right)}^{2}$$

This equation was derived for brittle materials, and it is uncertain if polymers follow the same relation. However, since this equation predicts the critical depth between ductile (i.e., plastic) deformation and brittle fracture, it may also be used for the assessment of the order of magnitude for polymers. However, when doing so, it becomes clear that in the case of polymers the values for this critical depth can vary between hundreds of microns and several centimetres. These values were corroborated by measurements and calculations presented by Aghababaei [40], as can be seen in Table 2.

This means that in the case of shallow wear and abrasion, the failure mechanism will always be ductile in polymers, and many brittle failure mechanisms such as lateral and median cracking will not occur. Lawn et al. [36] also conclude that in this case the volume of material removed from a material during grinding behaves according to “the long-standing Archard law”. So, even though Wu [38] has presented models to calculate the potential size of particles generated by the subsurface cracking during indentation and sliding of brittle materials, these are not applicable for ductile materials such as polymers for shallow wear (<100 μm) [41].

### 4.2. Archard Approach

Material removal at a depth below the critical indentation depth is conducted by ductile polishing/grinding. A simple estimate of the loss of material in this process is proposed by Archard [42] as:

(19) V=kPs

In which V is the loss in mm^3^, P the applied force in N and s the sliding distance in m. The specific wear rate coefficient k is material dependent and has the unit of mm^3^/Nm. A more elaborate derivation (Manoj [43], Salib [44]) starts with the observation that the area of contact in fully plastic wear is equal to πa^2^, where a is the radius of contact. The mean contact pressure in this case is related to the hardness of the softest, polymeric material:

(20) $$H=\frac{P}{{\pi a}^{2}}$$

After the contact asperity or indenter slides a length of 2a it is released from the contact and there is a probability K that this deformation forms a particle. The volume of such a particle is estimated to be a half sphere with radius a and volume 2πa^3^/3. The wear volume per sliding distance 2a then becomes W = K P/3 H. The total wear volume created by the indenter that is sliding over a distance of s can then be written as:

(21) $$V=\mathrm{Ws}=K\frac{P}{3H}s$$

Combining Equations (19) and (21) leads to the correlation between the empirical specific wear rate coefficient and the probability of debris formation: k = K/3H. Although Archard’s model was derived for adhesive wear, it has been used for other types of wear as well. This calculation is also valid if the contact area is not circular but square, e.g., in the case of a Vickers indenter or even for the grit on sanding paper [45].

### 4.3. Critical Length Scale of Adhesive Wear

For the final calculation of the number of particles generated by wear, it is required to obtain the size of the debris particles. According to Equation (20), the particle size that can be formed by plastic wear can go to infinitely small by reducing the normal force applied to the polymer. However, several authors have presented a critical length scale of adhesive wear below which no debris is released from the wearing surface. Aghababaei [46], Rabinowicz [45,47] and Ye [48] compared the stored elastic energy of a sliding interface (E_e_) with the adhesion energy (E_a_). The adhesion energy of a debris particle fully surrounded by polymer can be written as E_a_=W(πδ^2^). The elastic energy of a compressed particles with volume V can be expressed by E_e_ = σ^2^/2E*V = σ^2^πδ^3^/12E*. When E_e_ > E_a_, when the debris size is larger than a critical diameter, the debris will release itself from the surface. This approach was generated for adhesive wear, however, it can also be used for abrasive wear if the suitable material parameters are used: the effective modulus (E* = E/(1 − ν^2^)) and shear strength (σ = σ_S_) of the polymer that form the debris. Moreover, not only does the contact area of the hemispherical particles need to detach from the polymer surface, but the whole particle needs to break free. Then for E_a_ = E_e_ the critical debris size becomes:

(22) $$\delta_{W}=\frac{12\mathrm{EW}}{(1-\nu^{2})\sigma_{S}^{2}}$$

Which is similar to the Rabinowicz criterion [41,43], where W is the work of cohesion, which is the energy needed to separate a material into two parts and can be derived from the surface energy W = 2γ_Surf_ [46].

### 4.4. Microplastic Index from Wear

The MPI defined for impact in Equation (17) can also be calculated for wear. The volume of particles that are generated is given by the k factor of Equation (19) in m^3^/Nm. During wear, the load is perpendicular to the moving direction (=F_N_). The force in the direction of the movement (F_X_) is correlated with the normal load (=F_N_) via the coefficient of friction, μ: F_X_ = μF_N_. This results in a volume of particles produced by the input of wear energy as:

(23) $$V_{\delta W}=\mu k\;\;(m^{3}/J)$$

By combining the particle size, as estimated in Equation (22) with Equation (23), we can derive an equation for the number of particles formed:

(24) $$N_{\delta W}=\frac{\mu k}{\frac{1}{6}{\pi \delta }_{W}^{3}}\;\;(\#/J)$$

Similar to the impact MPI, the number of particles per joule can be normalized for a standard number of particles of N_REF_ = 1/J. The wear MPI then becomes:

(25) $${\mathrm{MPI}}_{W}=\log\left(\frac{N_{\delta W}}{N_{\mathrm{REF}}}\right)=\log\left(\frac{6\mu k}{{\pi \delta }_{W}^{3}}\right)$$

## 5. Determination of Material Properties

The MPI as presented for impact and wear in the previous sections can be used for the theoretical assessment of the tendency of polymers to form microplastics when an external stress is applied. This will also lead to the prediction of the theoretical particle sizes. For this calculation, several material properties are required:

- Critical stress intensity factor (K_IC_, MPa√m)
- Young’s modulus (E, GPa)
- Poisson’s ratio (ν)
- Coefficient of friction (μ)
- Yield strength (σ_Y_, MPa)
- Shear strength (σ_S_, MPa)
- Ball hardness (H, MPa)
- Specific wear rate coefficient (k, mm^3^/Nm)
- Ultimate tensile strength (σ_U_, MPa)
- Ultimate strain (ε_U_)
- Surface energy (γ, mN/m)
- Charpy notched impact (C_N_, J/cm^2^)

These parameters can be obtained experimentally, or taken from literature. For a first assessment of the relation between material properties and MPI, the calculation was executed for a series of polymers, for which the properties were taken from the literature. The website www.matweb.com [49] collects the properties of many materials and is a valuable source of polymer properties. When properties cannot be found on Matweb, alternative sources were used, such as The Polymer Handbook [50] and Properties of Polymers [51]. We have selected 14 polymers for the assessment of the Microplastic Index. However, many of these selected polymers are produced in various grades, giving rise to a broad range of property values. We have taken the average of many experimental results of many different grades to come to a general picture of the MPI for these polymers. An overview of the average values for the relevant properties for the selected polymers is given in the next Section. Unfortunately, three properties cannot be found reliably in the Matweb database: K_IC_, k, and σ_S_. The values for these parameters are collected from individual papers or derived from empirical or theoretical relations with more readily available parameters. K_IC_ is collected from 23 papers on the fracture of polymers [52,53,54,55,56,57,58,59,60,61,62,63,64,65,66,67,68,69,70,71,72,73,74,75]; k is related to the ultimate tensile strength and strain; and σ_S_ to the hardness and friction coefficient. The exact correlations to extract these parameters from measurements are explained in the next sections.

### 5.1. The Specific Wear Rate Coefficient

The specific wear rate coefficient, k, is given in mm^3^/Nm. This is the amount of debris that is formed by the application of a normal load along a specific length. It can also be assumed that k is the volume of particles formed by the application of a specific wear energy, and can be converted to m^3^/J. This value can be compared with the debris formed by impact as given by V_δ_ in Equation (15).

**Figure 3:**
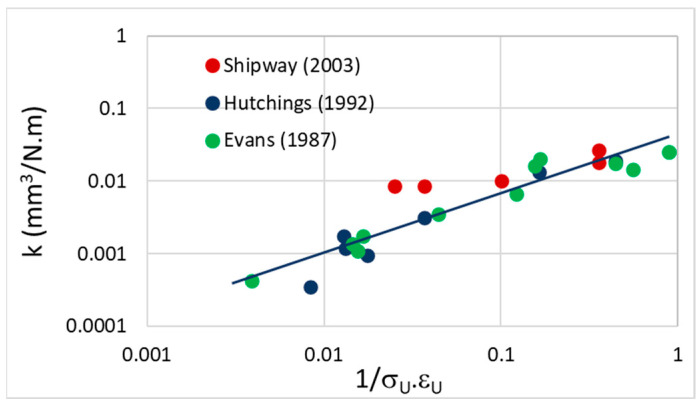
Specific wear plotted against the function of the ultimate stress and strain in a Lancaster-Ratner plot. Datapoints taken from Shipway [79]; Hutchings [80] and Evans [81].

The specific wear rate that is needed to calculate the release of particles by the friction between plastics depends heavily on the way the parameter is measured. This parameter is not widely available for many polymers, and needs to be recovered from literature. Lancaster [76], Ratner [77], Myshkin [78], and Shipway [79] present a relation between the wear coefficient and two material properties: the ultimate tensile stress (σ_U_) and the corresponding strain (ε_U_). The correlation between these parameters using data obtained from several references is shown in Figure 3 [79,80,81].

(26) $$\log\left(k\right)=0.8\log\left(\frac{1}{\sigma_{U}\varepsilon_{U}}\right)-1.34$$

In which σ_U_ (N/mm^2^) is the ultimate tensile stress and ε_U_ the corresponding strain (%). Therefore, e.g., low strength (small σ_U_) and low strain (small ε_U_) materials will generate more debris than more ductile materials. For most polymers the ultimate stress and strain can be found in databases such as Matweb.com. A detailed processing of the data can be found in the Table S2 (see in Supplementary Materials).

### 5.2. Shear Strength

The shear strength is the stress that is needed to shear the material and produce separated particles. This parameter is not often measured, but can be derived from the hardness [82]. The hardness is defined as shown in relation (20): H = P_N_/πa^2^. The stress at which the material starts to shear can be written as:

(27) $$\sigma_{S}=\frac{P_{S}}{A}$$

In which P_S_ is the shear force, and A the surface area (=πa^2^). The shear force is related to the normal force (P_N_) by the internal coefficient of friction, μ:

(28) $$P_{S}={\mu P}_{N}$$

This leads to the following estimation of the shear strength:

(29) $$\sigma_{S}=\mu H$$

## 6. Determination of Theoretical Particle Size and MPI for 14 Virgin Polymers

### 6.1. Material Properties

The assessment of a theoretical particle size and the MicroPlastic Index is demonstrated by the calculation of both impact MPI and wear MPI based on the literature values for polymer properties. For this calculation, the average values of the parameters of Tables S1 and S3 (see in Supplementary Materials) are used. The full list of properties and the literature used to derive them is shown in Table 3. In this table the shear strength is calculated from the hardness and the friction coefficient (Equation (29)), and the specific wear coefficient is calculated from the ultimate strength and strain (Equation (26)).

### 6.2. Impact Versus Wear

**Figure 4:**
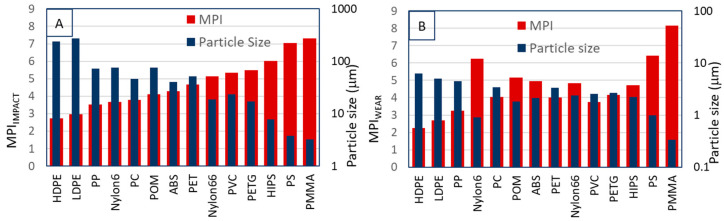
MPI_IMPACT_ (**A**), MPI_WEAR_ (**B**), and particle sizes plotted against the polymer type.

Using the parameters from Table 3, and the equations in the previous sections, we are now able to calculate the theoretical particle size and MPI for the impact and wear cases. The calculated values for the particle size and MPI are listed in Table 4 and shown in Figure 4.

The distribution in particle size and MPI for both cases has been calculated from the distribution in properties as listed in the Tables S1–S3 (see in Supplementary Materials). Two additional values of the MPI were calculated: one using all the minimal values; the other using all maximum values. These values were used to calculate the distribution in MPI and particle size. As we can see from Table 4, the difference in particle size and MPI between the various polymers is generally larger than the distribution within one polymer. This underpins the distinctive nature of the MPI for the comparison of the behavior of the various polymers.

Comparing the theoretical and experimental values for the size of the microplastics, there is a good correlation. Especially when we consider the standard deviations in both numbers and the fact that the theoretical prediction is based on the properties of many grades of polymers, and the experimental values only measured for one grade. Clearly the experimental grades are a subset of the selected polymer grades found in the literature databases.

Another observation for both theoretical and experimental results is that the particle size for impact is much larger than for wear. Similar particle sizes for impact (50–100 μm, [83]) and wear (1–5 μm, [84,85]) have been reported in the literature. The ratio of the largest particle (HDPE) and the smallest particle (PMMA) is much higher for impact (80) than for wear (10), indicating a different fracture mechanism, as has been shown in Section 3 and Section 4.

**Figure 5:**
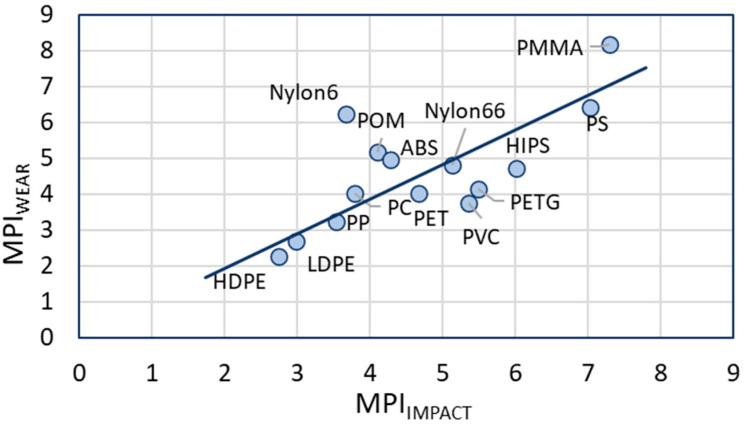
MPI_WEAR_ plotted against the MPI_IMPACT_ for the calculated values from Table 4.

Assessing the MPI, we notice that the MPI for impact is slightly higher than the MPI for wear. However, the overall order of the MPI of the polymers seems to be similar and the range of MPI is also comparable. The MPI_WEAR_ is plotted against the MPI_IMPACT_ in Figure 5.

The general trends for both impact and wear are the same for the whole set of polymers, with a few exceptions deviating from the fitted line in Figure 5. Some polymers show a higher tendency to form microplastics in impact than in wear (e.g., PVC and PETG), or vice versa (e.g., Nylon6). The polymer with the lowest tendency is HDPE, the polymers with the highest tendency are PS and PMMA. All these calculations show that the tendency of polymers to form microplastics are approximately similar for the impact and wear situation and can be predicted using the MPI approach, although some small deviations may occur. This creates the possibility to select the most appropriate polymers for applications in which the generation of microplastics is a risk.

It is important to note that the material properties used for these calculations are for virgin materials tested under standard conditions (room temperature, 50–60% RH and atmospheric pressure). This means that the estimations are representative for microplastic formation under these conditions; however, under other conditions they may be less representative. This may be of importance for predicting microplastic formation in specific environments, especially those with more extreme climates such as in polar, tropical or arid zones. Whilst the MPI models themselves do not include a temperature term, by using material properties determined under relevant conditions (temperature, pressure, humidity etc.), the influence of climate may be investigated.

The condition of the material also plays a large role in the tendency to form microplastics and the size of the formed microplastics. As mentioned above, these material properties are representative for virgin materials. However, we expect that recycled or degraded material will have different properties and therefore a different propensity to form microplastics. This is important as plastic that is in the environment will degrade over time leading to different material properties, and as such, a different MPI. The MPI we have determined for the 14 virgin polymers suggests that microplastics < 1 µm are rarely formed, although there is growing evidence in the literature that these nanoplastics are a bigger problem than previously thought. Polymer degradation could account for this difference. It may be that the properties of degraded plastics lead to an MPI suggesting smaller microplastics. In order to confirm this, further work is necessary to determine the effect of polymer degradation on material properties.

Finally, the MPI may also offer a route to develop solutions for the microplastic problem by helping to investigate replacement materials that form fewer microplastics. A simple example of this in effect would be the replacement of one grade of a plastic with another that forms less microplastics. It may also be possible to lower microplastic formation of a polymer by the addition of fillers and additives. An indication of the effect of this is seen by comparing the MPI for PS and HIPS in Figure 4 and Figure 5. HIPS is PS with the addition of rubber and it can be seen that the addition of rubber leads to a reduction in both the MPI and microplastic size formed.

## 7. Impact of MPI and Microplastics for the Global Environment

Besides the limited experimental validation on the size of formed microplastics, the assessment of the tendency of polymers to form microplastics is compared with literature data on microplastic presence in the global environment for 14 polymers [86,87,88,89,90,91,92,93,94]. The relative abundance of microplastics is plotted against the MPI for several environmental compartments (marine, fresh water and air) to investigate whether the predicted release of microplastics is also reflected in their environmental release.

Though many papers have been published, the values for plastic production and microplastic abundance vary tremendously. For the global production of polymers, ranges of values can be found depending on the market report. In addition, in most cases these production numbers have been published for a single year and for the assessment of the presence of microplastics, these numbers are required over a longer period of time. To correct for this, average growth rates have been derived from literature and used to extrapolate between the published years. For the amounts of microplastics in the environment, absolute values are even more difficult to obtain. The presence of different polymers has not always been published in full detail, and in many papers only the most abundant polymers are presented.

On top of that, the composition of the microplastics is heavily dependent on location, with respect to continents and type of compartment (sea, rivers and lakes, soil, air, etc.). The lack of standardized sampling and characterization techniques that are able to positively identify plastic particles over the entire micro- and nano-plastic range further add to the troubles of determining accurate environmental concentrations. However, a first attempt was made to gather indicative values for the different types of polymers by combining numerous literature reviews, each providing an overview of many papers on microplastics [95,96,97,98,99,100,101,102,103,104,105,106,107,108,109].

The environmental compartments assessed here include seas, rivers and lakes and air, and the number of data sources for each of these compartments is 247, 336 and 71 respectively. The collected data can be found in Tables S4 (seas), S5 (rivers and lakes) and S6 (air) (see in Supplementary Materials), and a summary of these data is given in Table 5. This table includes the distribution of the global production of polymers during the last 20 years. A detailed breakdown of global production can be found in Table S7 (see in Supplementary Materials). The investigated polymers contain various chemically similar plastics, i.e., HDPE and LDPE, Nylon 6 and Nylon 6.6, PET and PETG, and PS and HIPS. In many studies on microplastics, no differentiation is made between these similar polymers. To be able to use the individual polymer data, HIPS and PETG were not included in the microplastics composition study, since no paper could be found that differentiated these polymers, and the amounts expected in the environment are relatively low. The HDPE/LDPE and Nylon 6/Nylon 6.6 ratios in the microplastics are assumed to have a constant value, which is derived from a few studies that do differentiate, and from the ratio of these polymers in global production: HDPE/LDPE = 0.4/0.6, and Nylon 6/Nylon 6.6 = 0.66/0.34. The total amount of PET MPs also included polyester fibers, since PET is the major component. Table 5 only includes the MP found in sediment (sea), water and air, not in biota, because this may introduce an additional shift in composition.

The relative abundance of microplastics is calculated by dividing the % of a polymer in microplastics by the % of the global production of that polymer. If this number is 1, then the fraction of this polymer in microplastics is equal to the fraction of the global production.

**Figure 6:**
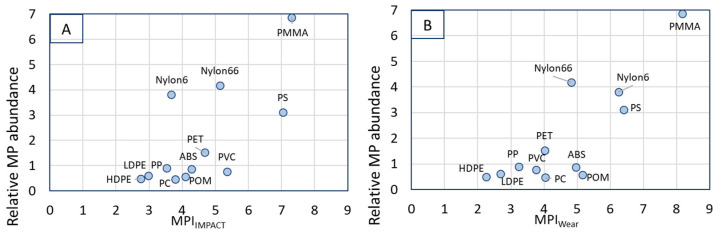

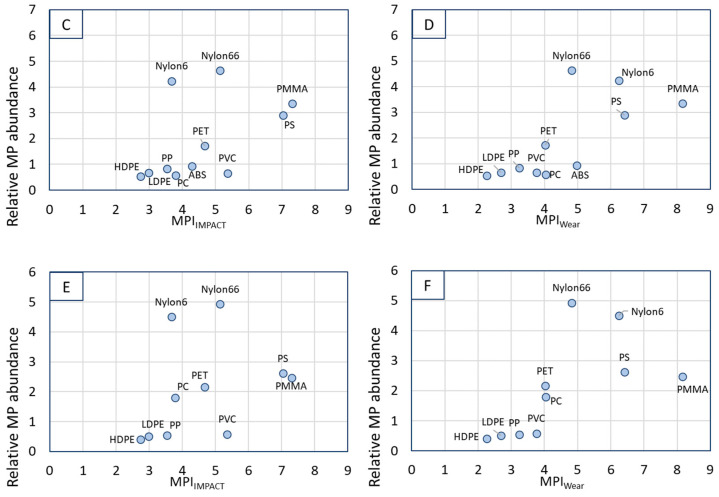
The relative abundance of microplastics in seas (**A**,**B**), in rivers and lakes (**C**,**D**) and in air (**E**,**F**) plotted versus the MPI for impact and wear.

When the composition of the microplastics in sea, rivers and lakes and air are compared, no significant differences can be found, although some polymers were not found in air in the references listed (e.g., ABS and POM). The important question is now, if these relative abundances of MPs can be correlated to the MPI calculated for these polymers. The data of Table 5 is plotted against the MPIs resulting in the graphs of Figure 6.

For all environmental compartments, the polymers with the lowest MPI have the lowest relative environmental abundance. There seem to be two outliers, the two polyamides (nylon 6 and nylon 6.6), that show an overestimation of the relative abundance, when plotted versus the MPI_IMPACT_. However, when plotted versus the MPI_WEAR,_ the two nylons seems to follow the trend better.

The correlation between MPI and microplastics found in air is poorer than for sea and fresh water, although in this case the relative presence of PS, PMMA and Nylon is also high.

## 8. Discussion & Conclusions

A novel method is derived for the calculation of microplastic formation of polymers based on their physical and mechanical properties. The MPI is calculated for impact versus wear (crack growth versus abrasion). Although the mechanics of particle formation are different for impact and wear, the overall MPI order of the polymers appears to be similar: HDPE and LDPE show the lowest MPI and PS and PMMA the highest (Figure 4). At the same time HDPE/LDPE has the largest particle size and PS/PMMA the smallest.

The first preliminary experiments on the formation of microplastics from four polymers by milling and sanding show that the experimental particle sizes of these polymers correspond well with the predicted values. Some of the calculated values exactly correspond to the predicted values, while others show more difference. Keeping in mind that the predicted values are derived from the properties of average polymers, and the tested polymers may have different properties, this agreement seems very promising for further exploration.

The MPI model and the experiments result in minimal sizes for the microplastics of around 1 μm for virgin polymers. When polymer properties change, e.g., due to ageing, sub-micrometer particles can be expected. However, this will only be valid when the properties of the polymers do not depend on the particle size. For particles smaller than 50–100 nm, the mechanical properties of polymer start to change [110,111] compared to bulk properties, and the MPI model will not be valid anymore.

There is a global correlation between the relative abundance of microplastics and the MPI_IMPACT_ and MPI_WEAR_ (Figure 6): the higher the MPI, the more abundant are the microplastics. This correlation appears to be better for the MPI_WEAR_ than for the MPI_IMPACT_. Some striking observations can be derived from Figure 6:

- Both Nylon6 and Nylon6.6 (mentioned as polyamide in many papers) are found as microplastics at a much higher concentration than expected from the MPI or the total polymer production. This is the case for all environmental compartments. This is most likely caused by the microplastics formed from fishing nets that are present in large abundancy in water environments. Globally, most fishing nets are made from nylon (46% followed by PET and PE (both ~20%) [112]. Degrading nets will add to the amount of polyamide in sea quickly, since it is already present. Since the correlation between of the Nylons with MPI_WEAR_ appears to be better, it could be concluded that wear is for this case the dominating fracture mechanism.
- PS and PMMA have the highest MPI and the highest abundancy in all environmental compartments. PS and PMMA are brittle, and a correlation between MPI and brittleness is evident.
- The presence of PMMA in marine environments is relatively higher than in fresh water. In the compiling of the compositions of microplastics in the different compartments, PMMA has been combined with acrylics that may be formed from ship paints. It is expected that the amount of paint microplastics is higher in sea than in rivers and lakes.
- PVC does not follow the trends in these plots. We have done the MPI calculations for rigid PVC. However, it can be expected that part of the PVC found as microplastics are plasticized plastics. This would mean that the impact strength, modulus and wear have significantly different values, shifting the predicted MPI to lower values.
- The correlation for microplastics in air is the poorest of the three, because the plastics found here are not only limited by composition but also by particle size and density that determine sedimentation from the air column. As a consequence, many polymers are not found in the air compartment.
- Assessing both the correlations for impact and wear, the actual reduction of plastic litter to smaller particles is most likely a combination of both. First the plastics breaks into large pieces by impact that further break up by wear and friction causes by wind and water.
- UV degradation due to environmental weathering is not included in the present study. It is expected that this will play a very important role in the formation of microplastics from plastics in the environment. This also means that the correlations we presented in Figure 6 are only an approximation.
- From the calculations and figures presented in this paper, it becomes clear that only PMMA, of the (virgin) polymers addressed, can form microplastics well below 1 μm.

The MPI calculations show that the particle size generated by wear is much smaller than the particle size generated by impact. Comparing the particle sizes calculated for impact and wear with those for the microplastics found in all papers assessed, it becomes obvious that the smallest wear particles (1–10 μm for most polymers) are much smaller than the microplastics formed by impact (>10 μm). This most likely means that wear particles are difficult to detect in environmental samples, and microplastic concentrations found in seas, rivers and lakes may be considered too low. Nevertheless, the correlation between MPI_WEAR_ and the relative abundancy of microplastics is relatively good, showing that larger particles also may be formed via wear mechanisms.

For the implementation of the MPI model, a full characterization of the polymer is needed. Although this may be laborious to execute, this also opens the possibilities of making or selecting adapted polymer grades (different random or block copolymers or molecular weights) that do not show a significantly difference in functional property, but do have a highly reduced microplastics formation risk. This will be the topic of future work, where we will experimentally validate the model in more depth and compare the MPI for a set of polymer grades, different processing conditions and ageing histories.

Textile washing, a relevant source of microplastics in wastewater, can be regarded as a combination of both impact and wear processes and will result in the formation microplastics with typical sizes as predicted by the MicroPlastic Index model. The degradation of these textiles in time will results in more and smaller microplastics formed.

A recent paper by Kärkkäinen and Sillanpää presented the results of some washing experiments of polyester and polyamide textiles and found the smallest fibers to be 30–100 µm for PES and 60–80 µm for PA, similar values to those calculated in Table 4 for these materials [113]. This indicates that the MPI model can also be implemented for textile materials.

We conclude that the MPI seems to be a very promising way of quantifying the microplastic formation from polymers. This MPI approach will be elucidated in future papers and validated with experimental results on different types and grades of polymers.

## Figures and Tables

**Table 1:** Experimentally measured particle sizes for impact (milling) and wear (sanding) fracture of four polymers.

	Impact	Wear
	δ (μm)	δ (μm)
HDPE	178 ± 59	5.2 ± 1.7
PP	75 ± 43	5.6 ± 1.8
PET	74 ± 44	2.6 ± 1.7
PS	3.1 ± 0.9	1.1 ± 0.4

**Table 2:** Comparing critical depth of fracture from literature and calculated from Equation (18).

Polymer	Ref [40]	Equation (18)
PMMA	0.13 mm	0.12 mm
HIPS	1.2 mm	0.95 mm
POM	2.1 mm	1.8 mm
PC	5.0 mm	3.6 mm

**Table 3:** Summary of the average polymer properties used in the calculation of the MicroPlastic Index. These values have been derived from the literature as listed in the last column. More elaborate tables are presented in the Supplementary Data.

	E GPa	H MPa	σ_Y_ MPa	σ_U_ MPa	σ_S_ MPa	ε_U_ %	ν	μ	Γ mN/m	C_N_ (J/cm^2^)	K_IC_ Mpa√m	k mm^3^/Nm	Refs
HDPE	1.04	49	26	26	13	638	0.44	0.27	35.7	2.0	3.6	8.8 × 10^−5^	[49,50,51,52,53,54,55,80]
LDPE	0.24	15	11	12	7	400	0.43	0.46	33.7	0.9	1.2	6.9 × 10^−5^	[49,50,51,56,57,81]
PP	1.47	68	32	38	17	188	0.43	0.25	29.6	3.6	2.4	3.2 × 10^−5^	[49,50,51,58,59,60,79,80,81]
PET	3.29	154	73	50	35	70	0.43	0.23	43.0	0.5	5	8.8 × 10^−4^	[49,50,51,59,61,62,81]
PETG	3.03	110	51	45	31	123	0.38	0.28	30.0	0.7	2.3	5.1 × 10^−4^	[49,50,51,63,79]
Nylon6	2.45	103	64	70	57	71	0.35	0.55	42.0	2.3	4.9	1.1 × 10^−3^	[49,50,51,64,65,81]
Nylon66	3.49	139	73	80	42	52	0.40	0.30	42.0	1.3	3.1	1.6 × 10^−3^	[49,50,51,59,66,80,81]
PVC	2.70	109	45	21	35	254	0.40	0.32	40.3	0.5	2.4	1.6 × 10^−4^	[49,50,51,55,59,61,67,79,81]
PMMA	2.91	171	64	64	100	10	0.39	0.58	40.2	0.3	1.1	5.0 × 10^−3^	[49,50,51,59,66,79,80,81]
PS	2.94	133	36	41	57	12	0.34	0.43	40.7	0.4	0.9	3.1 × 10^−3^	[49,50,51,59,67,79,80,81]
HIPS	1.99	81	26	27	33	45	0.41	0.40	40.7	1.0	1.3	7.7 × 10^−4^	[49,50,51,68,69]
PC	2.38	115	62	65	29	87	0.35	0.25	43.5	4.8	3.9	9.0 × 10^−4^	[49,50,51,59,66,67,70,71,81]
ABS	2.30	100	45	40	34	33	0.36	0.34	38.5	2.0	2.9	1.4 × 10^−3^	[49,50,51,72,73,81]
POM	3.01	161	67	63	43	37	0.37	0.27	41.5	0.9	5.5	1.8 × 10^−3^	[49,50,51,74,75,80,81]

**Table 4:** Calculated values for the particle size and MPI for impact and wear. The theoretical predictions are calculated according to Equations (10), (17), (22) and (25). The standard deviations are calculated from the distribution in the values as obtained from the literature databases (as shown in the Supplementary Data). The experimental results are taken from Section 2.

	Theoretical Prediction				Experimental Results	
	Impact		Wear		Impact	Wear
	δ (μm)	MPI	δ (μm)	MPI	δ (μm)	δ (μm)
HDPE	240 ± 19	2.7 ± 0.5	6.3 ± 0.3	2.3 ± 0.3	178 ± 59	5.2 ± 1.7
LDPE	272 ± 48	3.0 ± 0.3	5.0 ± 1.1	2.7 ± 0.3		
PP	72 ± 14	3.5 ± 0.1	4.4 ± 0.5	3.2 ± 0.3	75 ± 43	5.6 ± 1.8
PET	52 ± 8	4.7± 0.3	3.3 ± 0.3	4.0 ± 0.5	74 ± 44	2.6 ± 1.7
PETG	17 ± 8	5.5 ± 0.1	2.7± 0.6	4.1 ± 0.1		
Nylon6	76 ± 35	3.7 ± 0.2	0.9 ± 0.4	6.3 ± 0.1		
Nylon66	19 ± 11	5.1 ± 0.5	2.4 ± 1.3	4.8 ± 0.9		
PVC	24 ± 11	5.3 ± 0.2	2.6 ± 0.9	3.8 ± 0.3		
PMMA	3.2 ± 0.9	7.4 ± 0.4	0.3 ± 0.2	8.2 ± 0.7		
PS	3.8 ± 1.0	7.0 ± 0.3	1.0 ± 0.5	6.4 ± 0.1	3.1 ± 0.9	1.1 ± 0.4
HIPS	8 ± 3	6.0 ± 0.4	2.2 ± 0.1	4.7 ± 0.5		
PC	46 ± 11	3.8 ± 0.3	3.4 ± 1.4	4.0 ± 0.4		
ABS	41 ± 16	4.3 ± 0.2	2.1 ± 1.0	5.0 ± 0.5		
POM	75 ± 25	4.1 ± 0.1	1.9 ± 0.2	5.1 ± 0.6		

**Table 5:** Summary of the global production and microplastic composition of the polymers assessed.

	% of Global Production [86,87,88,89,90,91,92,93,94]	% of MPs in			Relative Abundancy (=% MP/% Global Production)		
		Sea [95,96,97,98,99,100,101]	Rivers and Lakes [9,102,103,104,105]	Air [106,107,108,109]	Sea	Rivers and Lakes	Air
HDPE	15.5	7.6	8.3	6.3	0.49	0.54	0.41
LDPE	18.7	11.4	12.4	9.4	0.61	0.66	0.50
PP	20.4	18.4	17.1	11.1	0.90	0.84	0.54
PET	9.9	15.0	17.0	21.3	1.53	1.72	2.17
PETG	-						
Nylon6	1.4	5.3	5.9	6.3	3.82	4.24	4.51
Nylon66	0.7	2.7	3.0	3.2	4.18	4.64	4.93
PVC	11.3	8.6	7.4	6.5	0.76	0.66	0.58
PMMA	1.0	6.8	3.3	2.4	6.86	3.35	2.47
PS	3.7	11.5	10.7	9.7	3.11	2.91	2.62
HIPS	-						
PC	1.1	0.5	0.7	2.0	0.47	0.58	1.80
ABS	2.4	2.1	2.2		0.87	0.93	
POM	0.5	0.3			0.57		
Rest	13.5	9.7	11.9	21.7			

## Data Availability

Most of the data used in this paper is derived from open literature and summarized in the Supplementary Data. The raw experimental data used for Figure 1 and Table 1 can be requested from the corresponding author.

## Supplementary Materials

The following supporting information can be downloaded at: https://www.mdpi.com/article/10.3390/polym15092185/s1, Table S1: Literature values for the Critical stress intensity factors of 14 polymers; Table S2: Literature data for the specific wear rate of several polymers; Table S3: Mechanical and physical properties of 14 polymers; Table S4: Global production of plastics (Mton/year); Table S5: Global composition of microplastics in marine environments; Table S6: Global composition of microplastics in fresh water environments (rivers and lakes); Table S7: Global composition of microplastics in air.
