# Supplementary material for: Microplastic Index—How to Predict Microplastics Formation?
Source: Polymers (Basel). 2023 May 4;15(9):2185. doi: 10.3390/polym15092185 (PMC10181052; doi:10.3390/polym15092185)
Supplement: Supplementary file 1 [file polymers-15-02185-s001.zip › polymers-2300502-supplementary.pdf]

polymers

Supplementary Materials: Microplastic Index – how to predict microplastics formation?

Arjen Boersma[1,*], Kalouda Grigoriadi [1], Merel G. A. Nooijens [1], Sieger Henke[2], Ingeborg M. Kooter[2], Luke A. Parker[2], Ardi Dortmans[2] and Jan Harm Urbanus[2]

Table S1. Literature values for the Critical stress intensity factors of 14 polymers.

Polymer	Range	K_{IC} (MPa√m)	REF
HDPE	Low	1.4	[52]
	High	5.9	[53]
	Average	3.6	[54], [55]
LDPE	Low	0.8	
	High	1.7	[56]
	Average	1.2	[57]
PP	Low	1.7	[58]
	High	3.9	[59]
	Average	2.6	[60]
PET	Low	2	[61]
	High	5.4	[59]
	Average	5.0	[62]
PETG	Low	1.8	[63]
	High	2.5	
	Average	2.3	
Nylon 6	Low	3.3	[64]
	High	5.5	[65]
	Average	4.9	
Nylon 6.6	Low	2.5	[59]
	High	3.9	[66]
	Average	3.1	
PVC	Low	2.0	[59]
	High	4.0	[55]
	Average	2.4	[67], [61]
PMMA	Low	0.7	[59]
	High	1.6	[66]
	Average	1.1	
PS	Low	0.7	[59]
	High	1.5	[67]
	Average	0.9	
HIPS	Low	1.0	[68]
	High	1.5	[69]
	Average	0.9	
PC	Low	3.0	[59], [67]
	High	5.1	[70]
	Average	3.7	[66], [71]
ABS	Low	2.5	[72]
	High	3.1	[73]
	Average	2.9	
POM	Low	4.4	[74]

	High	5.8	[75]
	Average	5.5	

Table S2. literature data for the specific wear rate of several polymers.

Polymer	$\sigma_U.\varepsilon_U$ (MPa.%) REF [79]	k (mm^3/N.m) REF [79]	$\sigma_U.\varepsilon_U$ (MPa.%) REF [80]	k (mm^3/N.m) REF [80]	$\sigma_U.\varepsilon_U$ (MPa.%) REF [81]	k (mm^3/N.m) REF [81]
PETG	27	0.0085				
PMMA	2.8	0.026				
PP	40	0.0085				
PS	2.8	0.018				
PVC	9.9	0.010				
PS			2.3	0.019		
PMMA			6.1	0.013		
POM			27	0.0031		
PP			76	0.0012		
PTFE			57	0.0010		
Nylon66			77	0.0017		
HDPE			119	0.0003		
PMMA					6	0.020
LDPE					256	0.0004
PS					2.3	0.018
POM					23	0.0035
Nylon66					60	0.0017
PP					69	0.0014
PTFE					64	0.0011
PET					1.8	0.015
PC					8.2	0.0067
ABS					6.4	0.016
PVC					1.1	0.025
Nylon6					47	0.0029

These values are derived from the graphs found in the literature, which may introduce a small error.

Table S3. Mechanical and physical properties of 14 polymers derived from [49,50,51].

Polymer	Range	E (GPa)	H (MPa)	σ_Y (MPa)	σ_U (MPa)	σ_S* (MPa)	ε_U (%)	C_N (J/cm^2)	ν	μ	γ (mN/m)
HDPE	Low	0.49	42	8	17	9	120	0.4	0.42	0.22	33
	High	1.53	58	40	44	17	1867	5.3	0.46	0.29	36.8
	Average	1.04	49	26	26	13	638	2.0	0.44	0.27	35.7
LDPE	Low	0.15	13	8	9	4	98	0.3	0.42	0.35	31
	High	0.41	16	21	36	10	783	1.7	0.44	0.65	35.3
	Average	0.24	15	11	12	7	400	0.9	0.43	0.46	33.7
PP	Low	0.75	56	19	13	11	8	1.1	0.43	0.20	29.4
	High	2.49	80	43	57	24	725	2.9	0.43	0.30	32
	Average	1.47	68	32	38	17	188	3.6	0.43	0.25	29.6
PET	Low	1.57	117	30	22	23	1	0.2	0.43	0.20	40
	High	5.20	170	90	95	51	600	1.4	0.43	0.30	44.6
	Average	3.29	154	73	50	35	70	0.5	0.43	0.23	43
PETG	Low	1.60	104	32	20	19	3	0.2	0.38	0.18	30
	High	4.20	121	59	100	39	360	1.0	0.38	0.32	30
	Average	3.03	110	51	45	31	123	0.7	0.38	0.28	30
Nylon 6	Low	1.10	90	38	44	27	3	0.4	0.23	0.30	40
	High	3.75	150	93	96	90	358	2.5	0.45	0.60	47
	Average	2.45	103	64	70	57	71	2.3	0.35	0.55	42
	Low	1.50	70	64	5	19	21	0.5	0.32	0.28	39.3

Nylon 6.6	High	1.85	180	91	138	50	210	2.3	0.42	0.28	46
	Average	3.49	139	73	80	42	52	1.3	0.40	0.30	42
PVC	Low	1.82	75	22	8	23	3	0.4	0.40	0.30	39
	High	4.04	130	55	57	52	465	0.5	0.40	0.40	41.5
	Average	2.70	109	45	21	35	254	0.5	0.40	0.32	40.3
PMMA	Low	1.86	173	55	46	78	3	0.2	0.37	0.45	39
	High	3.30	88	84	79	70	33	0.5	0.40	0.80	41.1
	Average	2.91	171	64	64	100	10	0.3	0.39	0.58	40.2
PS	Low	2.12	60	20	26	25	1	0.2	0.34	0.42	36
	High	3.48	155	56	56	71	70	0.8	0.34	0.46	42
	Average	2.94	133	36	41	57	12	0.4	0.34	0.43	40.7
HIPS	Low	0.75	60	30	12	24	1	0.1	0.41	0.40	36
	High	3.47	110	50	240	44	350	5.0	0.41	0.40	42
	Average	1.99	81	26	27	33	45	1.0	0.41	0.40	40.7
PC	Low	1.80	99	40	29	20	7	0.6	0.34	0.20	42.9
	High	3.24	125	112	90	46	186	8.9	0.38	0.37	45
	Average	2.38	115	62	65	29	87	4.8	0.35	0.25	43.5
ABS	Low	1.40	68	34	12	20	2	0.4	0.36	0.30	38.5
	High	2.83	115	65	74	55	130	9.5	0.38	0.48	38.5
	Average	2.30	100	45	40	34	33	2.0	0.36	0.34	38.5
POM	Low	1.93	150	45	45	38	2	0.4	0.37	0.25	38
	High	3.72	175	90	76	53	110	1.6	0.37	0.30	45
	Average	3.01	161	67	63	43	37	0.9	0.37	0.27	41.5

* Calculated from other parameters

Number of data points taken from the literature [49,50,51] to calculate the minimum, maximum and average values for the properties listed in the table above.

Range	E (GPa)	H (MPa)	σ_Y (MPa)	σ_U (MPa)	ε_U (%)	C_N (J/cm²)	ν	μ	γ (mN/m)
HDPE	171	42	904	408	759	78	3	12	3
LDPE	194	3	314	323	362	3	2	214	2
PP	115	68	1402	292	566	76	1	32	3
PET	56	9	46	35	61	26	1	12	2
PETG	39	3	48	62	64	17	1	3	1
Nylon 6	509	88	497	324	659	380	7	78	2
Nylon 6.6	335	13	307	294	437	290	48	19	2
PVC	425	4	623	1194	1243	9	1	4	3
PMMA	277	32	72	289	339	83	9	6	2
PS	159	18	149	168	228	37	1	3	3
HIPS	289	24	376	268	436	83	1	1	2
PC	484	84	679	391	653	213	9	7	2
ABS	416	79	774	385	607	340	5	2	1
POM	82	5	103	40	126	64	21	15	2

Table S4. Global composition of microplastics in marine environments (in %).

	REF [95]** Global 2012	REF [96]** Global Beach 2022	REF [96]** Global Water 2022	REF [96]** Global Sediment 2022	REF [97]** Africa 2021	REF [98] Global 2022	REF [99] Pacific 2020	REF [100] Mariana Trench 2018	REF [101] Polar sea 2021	Average
Number of studies	68	30	55	26	34	11	18	3	2	
HDPE	11.9	7.5	6.4	6.0	8.3	5.6	6.3		4.6	7.6
LDPE	17.9	11.3	9.6	8.9	12.5	8.4	9.4		6.9	11.4

PP	24.2	19.5	15.6	14.9	18.2	10.8	39.1	1.7	4.5	18.4
PET	4.5	10.1	10.5	9.9	10.4	26.0	14.2	14.4	32.5	9.2
PETG			PETG microplastics cannot be distinquished from PET microplastics							
Nylon6	4.2	7.2	7.7	6.0	4.3	6.1		7.1	2.3	5.3
Nylon66	2.2	3.7	3.9	3.1	2.2	3.1		3.7	1.2	2.7
PVC	9.4	10.1	9.1	10.8	7.8	6.0		22.4	9.5	8.6
PMMA	5.7		7.6	7.4	3.9	11.8	16.2		10.0	6.8
PS	15.1	12.3	9.8	11.6	18.2	7.0	5.3	13.1	6.0	11.5
HIPS			HIPS microplastics cannot be distinquished from PS microplastics							
PC		0.7	0.4			1.3			1.0	0.5
ABS		2.9	1.5	2.5	2.6			4.1	3.0	2.1
POM	0.8	0.0							1.0	0.3
PUR	0.4	2.9	3.6	2.5	1.3	4.5		11.7	5.5	2.1
PEST		5.8	7.3	9.9	2.6			8.1		5.9
Rest	3.7	6.0	7.0	6.5	7.7	9.4	9.5	13.7	12.0	7.6

** The microplastics in these papers are not presented as concentrations of the total, but as number of papers in which the specific polymer is found. This data is converted to percentages by dividing the number of papers having microplastics of the specific polymer by the total number of papers that contain all microplastics.

Table S5. Global composition of microplastics in fresh water environments (rivers and lakes, in %).

	REF [102] Developed countries 2020	REF [102] Developing countries 2020	REF [103] Global 2020	REF [9]** Global 2019	REF [104] Global Water 2021	REF [104] Global Sediment 2021	REF[105] Switzerland	Average
Number of studies	18	19	28	32	127	107	5	
HDPE	16.3	7.8	9.6	7.1	8.3	8.3	12.3	8.3
LDPE	24.5	11.8	14.4	10.6	12.5	12.5	17.7	12.5
PP	17.7	23.6	24	16.4	18.2	15.9	21.2	17.2
PET	3.5	29.8	11	8.2	19.2	2.2	8.4	11.1
PETG								-
Nylon6		4.4	4.0	5.0	4.9	9.0	7.1	5.9
Nylon66		2.2	2.0	2.6	2.5	4.7	3.7	3.0
PVC	4.0	1.7	1	8.9	14.0	3.4	14.3	7.4
PMMA				3.1	0.8	6.8	4.1	3.3
PS	13.9	9.1	13	12.0	14.0	7.9	3.1	10.8
HIPS								-
PC				0.6			1.2	0.7
ABS	2.0			1.9			2.4	1.9
POM								0.0
PUR	0.9		1.0	2.5	0.5	6.8	4.6	2.8
PEST	1.8	2.3		15.9	1.9	10.3		6.0
Rest	15.4	7.3	20	5.2	3.2	12.2	0	9.1

Table S6. Global composition of microplastics in air (in %).

	REF [106] Asia 2022	REF [106] Europe 2022	REF [107] Atlantic coast 2020	REF [108]** Global Outdoor 2021	REF [108]** Global Indoor 2021	REF [109]* Global 2022	Average
Number of studies	8	6	4	27	8	18	

HDPE	5.3	3.9	25.5	5.7	5.3	5.8	6.3
LDPE	7.9	5.8	38.2	8.5	8	8.8	9.4
PP	9.4	6.9	5.4	10.8	16.7	14.6	11.1
PET	34.0	20.3	9.6	8.3	13.3	15.6	14.0
PETG							-
Nylon6	9.0	6.5		5.5	8.8	6.2	6.3
Nylon66	4.6	3.3		2.8	4.5	3.2	3.2
PVC	11.7	7.2	9.5	7.5	6.7	3.1	6.5
PMMA				3.3	3.3	1.0	2.4
PS	7.2	21.1	11.8	11.7	10	5.2	9.7
HIPS							-
PC				0.8	6.7	2.1	2.0
ABS							-
POM							-
PUR	1.2			0.8		2.1	1.3
PEST				10	10	3.1	7.3
Rest	9.7	25.0	0	24.3	6.7	29.2	16.5

Table S7. Global production of plastics (Mton/year).

	1990	1995	2000	2005	2010	2015	2020	Total*	%	REF
HDPE	17.3	22.3	30.0	35.9	43.6	51.6	59.6	260	15.5	[86]
LDPE	19.5	26.3	35.2	45.7	52.2	63.7	73.0	316	18.7	[86]
PP	23.2	29.4	38.6	48.5	55.9	68.0	80.1	344	20.4	[86]
PET	10.5	14.2	19.5	22.6	27.8	33.1	38.3	166	9.9	[86]
PETG	0.1	0.2	0.2	0.3	0.3	0.4	0.5	2	0.1	[87]
Nylon6	1.4	1.8	2.3	3.0	3.8	4.9	6.2	23	1.4	[88]
Nylon66	0.7	0.9	1.1	1.4	1.8	2.3	2.9	11	0.7	[89]
PVC	12.7	15.8	21.6	27.8	30.6	37.4	44.2	190	11.3	[86]
PMMA	0.9	1.3	1.8	2.5	2.9	3.2	4.0	17	1.0	[90]
PS	3.1	4.2	5.6	7.5	10.1	13.5	18.1	62	3.7	[91]
HIPS			No market data could be found							
PC	0.8	1.2	1.9	2.9	3.5	4.1	4.6	19	1.1	[92]
ABS	1.3	2.0	3.1	4.6	7.0	10.6	11.4	40	2.4	[93]
POM	0.5	0.6	0.8	1.0	1.3	1.7	2.1	8	0.5	[94]
REST	12.7	18.9	24.7	30.6	37.4	46.4	55.3	226	13.4	[86]

*is the sum of the polymer produced in the years 1990, 1995, 2000, 2005, 2010, 2015 and 2020.
